# Unintentional Epinephrine Auto-Injector Maxillofacial Injury in a Pediatric Patient

**DOI:** 10.5811/cpcem.2021.11.54464

**Published:** 2022-01-15

**Authors:** Jason David, Jerad Eldred, David Raper

**Affiliations:** * Nellis Air Force Base, Mike O’Callaghan Military Medical Center, Department of Emergency Medicine, Las Vegas, Nevada; † University of Nevada, Las Vegas, University Medical Center of Southern Nevada, Department of Emergency Medicine, Las Vegas, Nevada

**Keywords:** epinephrine auto-injector, EpiPen, discharge, maxillofacial, pediatrics

## Abstract

**Case Presentation:**

A four-year-old female patient presented to the emergency department with an epinephrine auto-injector that had unintentionally discharged into her mandible. There was difficulty removing the auto-injector at bedside. Images we acquired noted needle curvature not present in an off-the-shelf model. She was sedated, and the auto-injector was removed by retracing the angle of discharge, with care taken not to inject epinephrine into the patient.

**Discussion:**

Epinephrine auto-injector accidental discharges are an unusual injury pattern, but the incidence of such events is increasing in the United States. The emergency clinician should be cognizant of complicating factors with discharges, such as bent needles. Here we discuss a case of discharge into the maxillofacial region (lower jaw), with approaches to treatment.

## CASE PRESENTATION

**Image 1:**
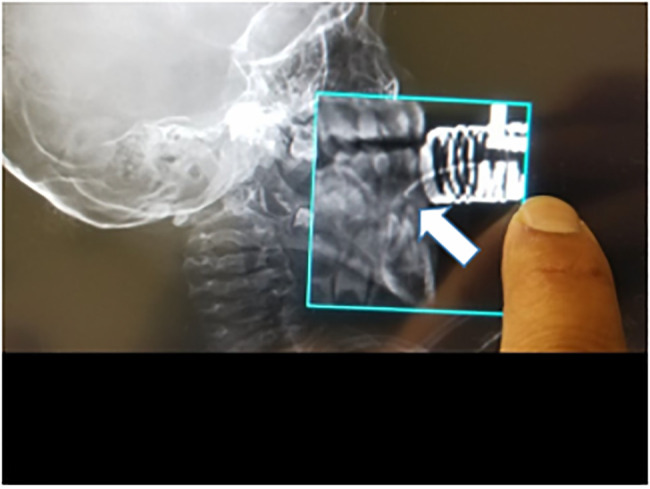
Cranial radiographs identifying foreign body (arrow).

**Image 2:**
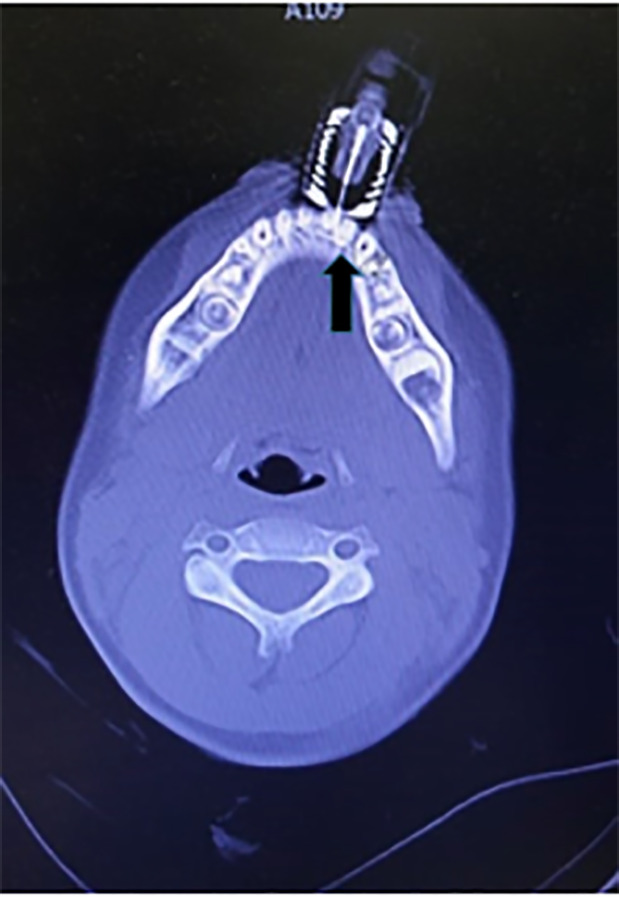
Maxillofacial computed tomography with axial view reconstruction demonstrating hooked foreign body (arrow).

A four-year-old female presented to the emergency department (ED) with an epinephrine auto-injector unintentionally injected and lodged in her lower jaw that entered through the gingiva. Radiographs of the skull and computed tomography (CT) revealed a hooked epinephrine auto-injector embedded between the lower central incisors beneath the gingival line, bent at an approximately 140° angle (Images 1 and 2).

**Image 3:**
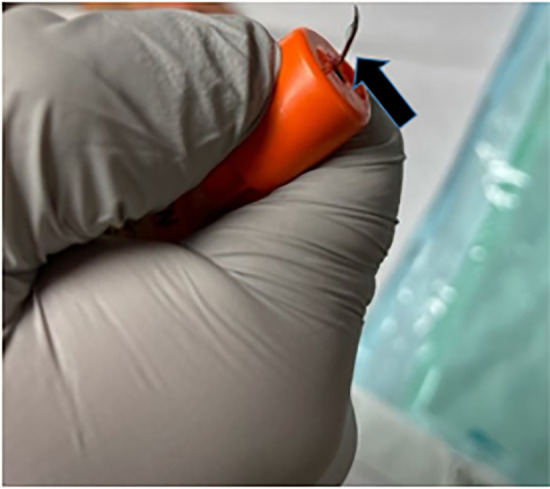
Removed epinephrine auto-injector and bent needle (arrow).

An initial attempt was made by the parents to remove the object at home followed by an attempt at ED bedside, which proved to be difficult due to needle angulation. We consulted oral-maxillofacial surgery; the patient was sedated with intravenous ketamine, and under sedation the hook was pulled in a retrograde manner following the noted posterior-lateral trajectory of the bent needle tip on maxillofacial imaging (Image 3). With some force, the needle was removed with care that it not accidentally discharge epinephrine into the patient. There was a very minor avulsion of gingival mucosa, which we learned did not interfere with patient’s oral intake or speech after following up with parent.

## DISCUSSION

Unintentional epinephrine auto-injector injuries typically occur in the digits or the legs.^1^ These cases are on the rise, as epinephrine auto-injectors have become more commonly prescribed.^2^ However, exploring the world by placing objects in their mouths is a normal stage of early childhood development.^3^ This case was particularly concerning due to the initial difficulty in removing the auto-injector and fear of accidentally discharging the adult-dose epinephrine into the patient. Efforts were made to stabilize the auto-injector with a bulky dressing and pillow. Due to the difficulty with initial removal, imaging was pursued. Maxillofacial CT is the optimal imaging study.^4^

**CPC-EM Capsule**

What do we already know about this clinical entity?

*Epinephrine auto-injector accidental discharges are a unique and rising injury pattern in the United States and can have deceivingly simple presentations*.

What is the major impact of the image(s)?

*Emergency medicine physicians should be aware of complicating factors with accidental auto-injector injuries, such as bent needles, and appreciate nuances to treatment*.

How might this improve emergency medicine practice?

*Emergency medicine physicians will be more familiar with this particular injury pattern and be more effective at treating similar auto-injector injuries*.

The mechanism behind the hooking of the needle of the auto-injector could presumably be due to hitting the subgingival areas of the incisor and curving, as well as bending, during attempted removals. Postulations from similar case studies regarding the curvature of auto-injector needles include bending when hitting a bone during injection, bending when the patient moves during injection, or if the needle fires off center and hits the cartridge carrier, hooking the needle prior to injection.^5^ This situation should be anticipated and investigated with imaging by the treating physician before attempting to remove the needle blindly. Stabilizing the auto-injector with a pillow and bulky dressing will also prevent further bending of the needle, which would make removal more difficult. Furthermore, care should be taken not to accidentally discharge the epinephrine dose, either by securing the pen with a bulky dressing as we did, or by removing the chamber (which we found to be extremely difficult). Prudent emergency physicians should keep these factors (object stabilization, needle curvature, and remaining epinephrine dose) in mind when treating a victim of accidental auto-injector discharge to a sensitive area such as the face.
